# Lessons Learned from Rwanda: Innovative Strategies for Prevention and Containment of COVID-19

**DOI:** 10.5334/aogh.3172

**Published:** 2021-02-25

**Authors:** Naz Karim, Ling Jing, J. Austin Lee, Ramu Kharel, Derek Lubetkin, Camille M. Clancy, Doris Uwamahoro, Ernest Nahayo, Joseph Biramahire, Adam R. Aluisio, Vincent Ndebwanimana

**Affiliations:** 1 Department of Emergency Medicine, Brown University Alpert Medical School, Providence, USA; 2 Department of Anesthesia, Critical Care, and Emergency Medicine, College of Medicine and Health Sciences, University of Rwanda, Kigali, Rwanda; 3 Department of Emergency Medicine, Kigali University Teaching Hospital (CHUK), Rwanda; 4 Covid19 treatment center, Rwanda Military Hospital, Rwanda

## Abstract

**Introduction:**

Rwanda has made significant advancements in medical and economic development over the last 20 years and has emerged as a leader in healthcare in the East African region. The COVID-19 pandemic, which reached Rwanda in March 2020, presented new and unique challenges for infectious disease control. The objective of this paper is to characterize Rwanda’s domestic response to the first year of the COVID-19 pandemic and highlight effective strategies so that other countries, including high and middle-income countries, can learn from its innovative initiatives.

**Methods:**

Government publications describing Rwanda’s healthcare capacity were first consulted to obtain the country’s baseline context. Next, official government and healthcare system communications, including case counts, prevention and screening protocols, treatment facility practices, and behavioral guidelines for the public, were read thoroughly to understand the course of the pandemic in Rwanda and the specific measures in the response.

**Results:**

As of 31 December 2020, Rwanda has recorded 8,383 cumulative COVID-19 cases, 6,542 recoveries, and 92 deaths since the first case on 14 March 2020. The Ministry of Health, Rwanda Biomedical Centre, and the Epidemic and Surveillance Response division have collaborated on preparative measures since the pandemic began in January 2020. The formation of a Joint Task Force in early March led to the Coronavirus National Preparedness and Response Plan, an extensive six-month plan that established a national incident management system and detailed four phases of a comprehensive national response. Notable strategies have included disseminating public information through drones, robots for screening and inpatient care, and official communications through social media platforms to combat misinformation and mobilize a cohesive response from the population.

**Conclusion:**

Rwanda’s government and healthcare system has responded to the COVID-19 pandemic with innovative interventions to prevent and contain the virus. Importantly, the response has utilized adaptive and innovative technology and robust risk communication and community engagement to deliver an effective response to the COVID-19 pandemic.

## Background

Coronavirus Disease (COVID-19), caused by the severe acute respiratory syndrome coronavirus 2 (SARS-CoV-2), which originated in Wuhan, China in late 2019, has spread across the globe, rapidly reaching classification as a pandemic. As of 12 January 2021, more than 89 million cases and nearly 2 million deaths have been identified worldwide and nearly every country has been impacted [1].

According to the World Health Organization (WHO) surveillance data, the African region (AFRO) has 2,172,862 confirmed cases and 24,464 deaths from COVID-19 as of 12 January 2021 [1,2]. Unique socioeconomic and health aspects in the African context present challenges to identifying and treating COVID-19 cases, including low relative per-capita health resources, a rapidly growing population, and multiple endemic infectious diseases [3,4]. Despite these challenges, many countries have implemented rapid, aggressive interventions that may help explain the milder pandemic course in Africa than in other world regions [5].

Rwanda is an East African nation of more than 12 million people, with 83% of the population living in rural areas [6]. The country has made significant advancements in healthcare and economic development over the last 20 years and has emerged as a leader in healthcare in the East African region. Rwanda operates under a universal healthcare model where public health insurance coverage is nearly 84%, with another 6% of the population covered through other insurance policies [7]. The country has approximately 1,350 doctors, 9,551 nurses, and 21,826 hospital beds for its 30 districts, for an average of 8,919 people per doctor, 1,261 people per nurse, and 552 people per bed [8]. Per district, the population per doctor ranges from 1,725 in Nyarugenge to 54,266 in Nyaruguru. The population per nurse ranges from 412 in Nyarugenge to 3,256 in Nyaruguru, and the population per bed from 288 in Nyarugenge to 1,025 in Nyagatare located in the Eastern Province [8]. In comparison, the global averages for population per doctor, nurse, and bed are 666, 294, and 370, respectively. The averages in Sub-Saharan Africa for population per doctor, nurse, and bed are 5000, 1000, and 833, respectively [9]. Rwanda has eight national referral hospitals, four provincial hospitals and 36 district hospitals [7]. Locally, there are around 45,000 community health workers working at 504 health centers and 670 health posts, providing the population further access to the health system [7,10,11].

While African countries have set up the Africa Task Force for Coronavirus Preparedness and Response (AFTCOR) in concert with the African Union Commission, the Africa Centres for Disease Control and Prevention (Africa CDC), and the WHO [6], the Rwandan government has also taken an engaged and innovative approach to COVID-19. As part of its COVID response, Rwanda announced the formation of a Joint Task Force on 3 March 2020, which created the Rwandan Coronavirus National Preparedness and Response Plan, with the primary objective of “stopping the human-to-human transmission of the virus and caring for those affected [12].”

## Overview of the COVID-19 Response in Rwanda

The Rwandan government has utilized creative strategies to prevent and contain COVID-19, guided by an extensive six-month National Preparedness and Response Plan that details four focused phases of response, including establishment of a national incident system [12]. Innovative strategies include remote case identification, use of a toll-free hotline, a national WhatsApp™ number, drones for information dissemination, and robots for patient monitoring in hospitals. Robust risk communication and community engagement plans using social media platforms have also helped combat misinformation and increase public knowledge around COVID-19.

### Availability of Equipment, Testing, and Capacity

Due to the transmissibility and infectivity of the SARS-CoV-2 pathogen, there is a crucial need for high screening and testing capacity. COVID-19 case management can require significant health resources in patients with severe or critical illness, which can be challenging in Rwanda, where there is limited ICU capacity [13]. To preemptively address the issue of capacity, the government of Rwanda has allocated funds to the development of COVID-19 health units, purchased equipment for testing, and installed thermal imaging cameras to screen all arrivals at air and land borders [12,14]. Personal protective equipment (PPE) and testing kits have been purchased or donated by different stakeholders. Tests obtained at hospitals are analyzed by the National Reference Lab. In addition to these preventative and containment measures, confirmed cases are admitted, and their contacts are followed for isolation, or tested if they have had any symptoms during the last 14 days. This is done at no cost to the population, as many are unable to afford these measures, and requiring payment would threaten the safety of individuals and the efficacy of containment.

Demonstrating the flexibility of the Rwandan health system, critical infrastructure utilized in combating COVID-19 was based on existing built structures. In February 2020, the Kanyinya health center initially constructed in 2013 was transformed into a COVID-19 treatment center with 75 floor beds and 8 ICU beds that can manage patients in isolated units. Following this model, a second COVID-19 treatment center with 126 floor beds and 8 ICU beds was opened two weeks later by repurposing a hotel. Additionally, each of the country’s 80 public hospitals were requested to set aside two beds for the isolation of patients under investigation and COVID-19 treatment. In early May, to further protect health care staff and minimize transmission risks, treatment centers began using robots for patient monitoring and care documentation [15]. Treatment teams have been assembled to ensure the holistic medical and psychological welfare of patients. Each unit includes a medical director, doctors, nurses, clinical psychologist, biomedical staff, nutritionists, infection prevention control (IPC), administrators, logistic managers, and data managers.

### Current Public Health Response

**Figure 1:**
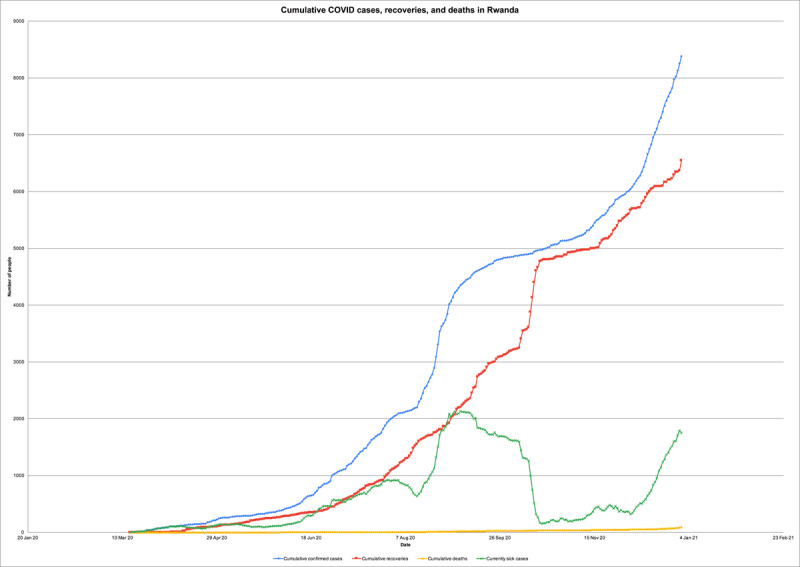
Confirmed COVID-19 cases, recoveries, and deaths in Rwanda.

Rwanda reported its first confirmed COVID-19 case, a returning traveler, on 14 March 2020 [16]. As of 31 December 2020, Rwanda has performed more than 730,000 tests and recorded 8,383 cumulative cases, 6,542 recoveries, and 92 deaths (***Figure 1***) [17]. Confirmed cases are immediately isolated and followed up with contact tracing [16]. Around 90% have been local cases, with incoming travelers and their contacts comprising the remainder [17]. Men constitute approximately 65% of the country’s cases [14]. Most of those tested have been asymptomatic or in stable, non-critical conditions [16].

The Rwandan Ministry of Health (MOH) has rapidly implemented additional innovative public health strategies to respond to the pandemic. These strategies include creating guidelines and forming expert teams to coordinate and organize the response plan; providing accurate, timely, and thorough information to the public; enforcing national prevention guidelines; and establishing designated treatment centers for COVID patients. Though challenges remain, the combination of these strategies has likely contributed to the prompt and effective containment of COVID in Rwanda thus far.

**Figure 2:**
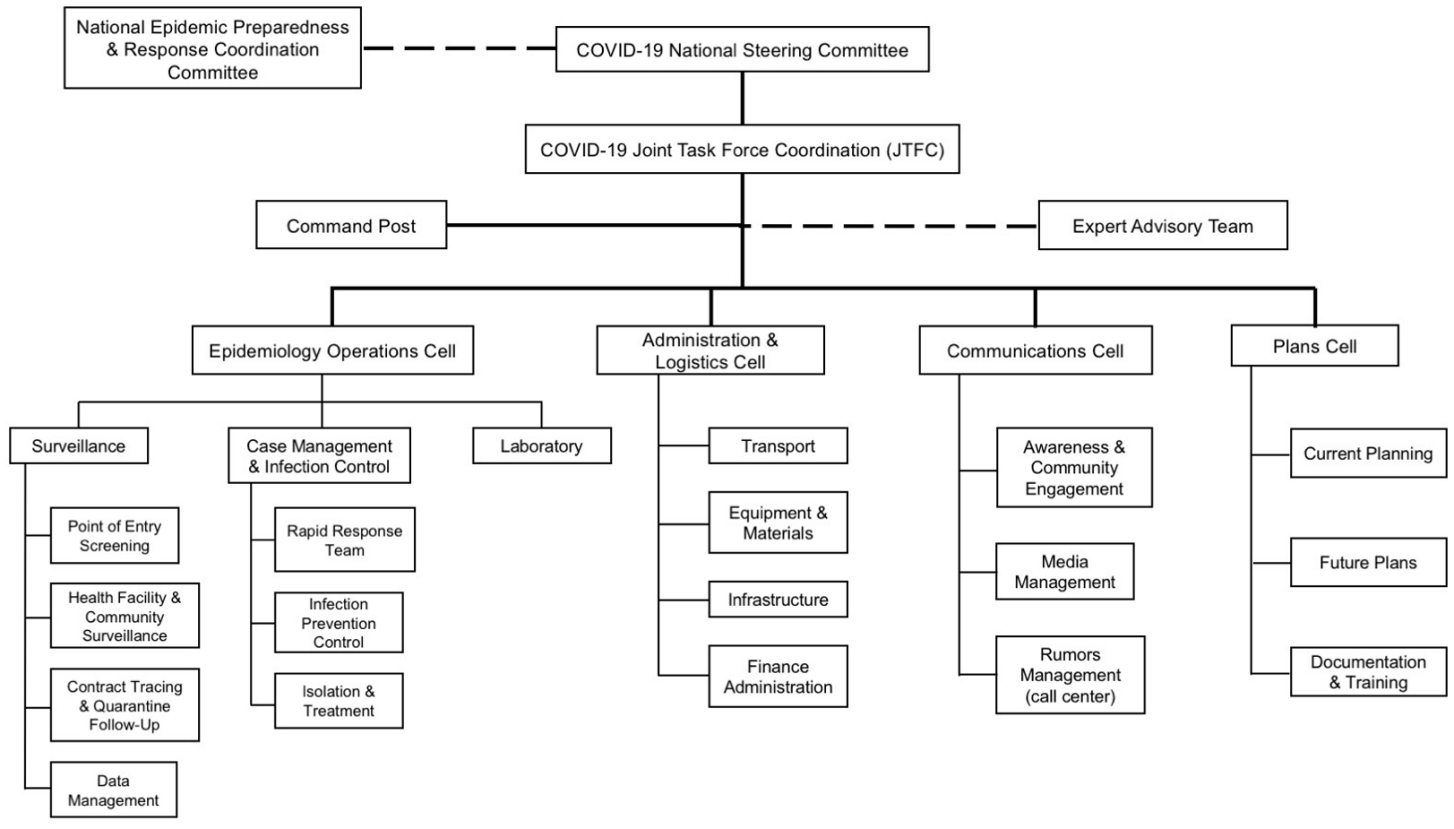
Rwandan Government COVID-19 Response Infrastructure.

The government of Rwanda has activated the National Epidemic Preparedness and Response Coordination Committee, which appointed the COVID-19 National Steering Committee to oversee the coordination of Joint Task Force activities, grouped into epidemiology operations, administrative and logistics communication, and planning units (***Figure 2***) [12]. The Rwanda Biomedical Centre (RBC) and the MOH are the nation’s central health implementation agencies who have collaborated to develop a six month National COVID-19 Preparedness and Response Plan [12]. This plan includes four phases of country response: Pre-epidemic; sporadic cases dealing with limited numbers of imported cases from affected countries; single clusters of cases responding to local transmission in a district, sector, or village; and community transmission involving more than one cluster [12]. The COVID-19 response in each of Rwanda’s 30 districts is led by the mayors, who coordinate the interventions for every aspect of infection control [18]. Much of Rwanda’s pandemic response has adopted or leveraged existing infrastructure from Ebola preparedness efforts in 2018–19, highlighting the advantages of comprehensive pandemic preparation experience for a country [19,20]. For example, strategies for developing a National Preparedness Plan, training health workers and equipping health facilities, establishing dedicated treatment centers, conducting simulation exercises, educating the public, and screening extensively at national points of entry during the Ebola efforts have served as a strong foundation for the COVID-19 response [19,20].

During the preparation phase, incident management concentrated on establishing a rapid response team (RRT) in districts and health units, with 480 trained staff, in addition to the creation of COVID-19 Standard Operating Procedures (SOPs) [21]. The SOPs guide the national response to COVID-19, including leadership structure, infection prevention, epidemiological surveillance, handling of clinical specimens, psychosocial support, case management, community mobilization, and managing misinformation. Importantly, the plan includes the critical step of forming a coordinated national COVID-19 Incident Management System and outlining the allocation of $73,471,760 USD to be utilized over the next 6 months [12].

RBC recommendations for healthcare facilities include adequate triage, early recognition of patients under investigation with subsequent isolation, and ultimately, transfer to a COVID-19 treatment center if necessary. They also discuss the need for PPE training and hand-washing stations at all health facilities’ points of entry [21]. Their recommended criteria for discharge from COVID-19 treatment centers after 14 days includes three or more afebrile days, significant improvement in clinical condition, and two negative PCR tests more than 24 hours apart [21]. Additionally, the RBC highlights the importance of patients receiving appropriate psychological counseling prior to discharge; the psychosocial team is informed once the first test has resulted in order to provide services without delaying discharge [21].

The MOH and RBC have released guidelines for community health workers to implement contact tracing [21]. They recommend a protocolized system of sharing information related to active cases through Rwanda’s Electronic Infectious Disease Surveillance and Response (eIDSR) and the National Reference Laboratory [21]. The SOP provides a stepwise process for discovering and combating potentially harmful misinformation through daily media review and a coordinated multi-media response [21]. The RBC and MOH also outline an organized effort to address COVID-19 related psychological issues through interventions and mental health education specifically designed for health-care workers, patients, and the general population. The National Epidemic Prevention Control Coordination Committee (NEPCCC) is tasked with carrying out the recommended psychological intervention to reduce psychological damage and encourage social stability [21]. The critical need for continued services to treat critical non-COVID-19 public health needs, including HIV, TB, malaria, and maternal health is emphasized. Of note, they plan to distribute 2–3 months of antiretroviral therapy for known HIV patients [12]. They will also utilize trained community health workers to complete mass distribution of long-lasting insecticidal nets [12].

**Figure 3:**
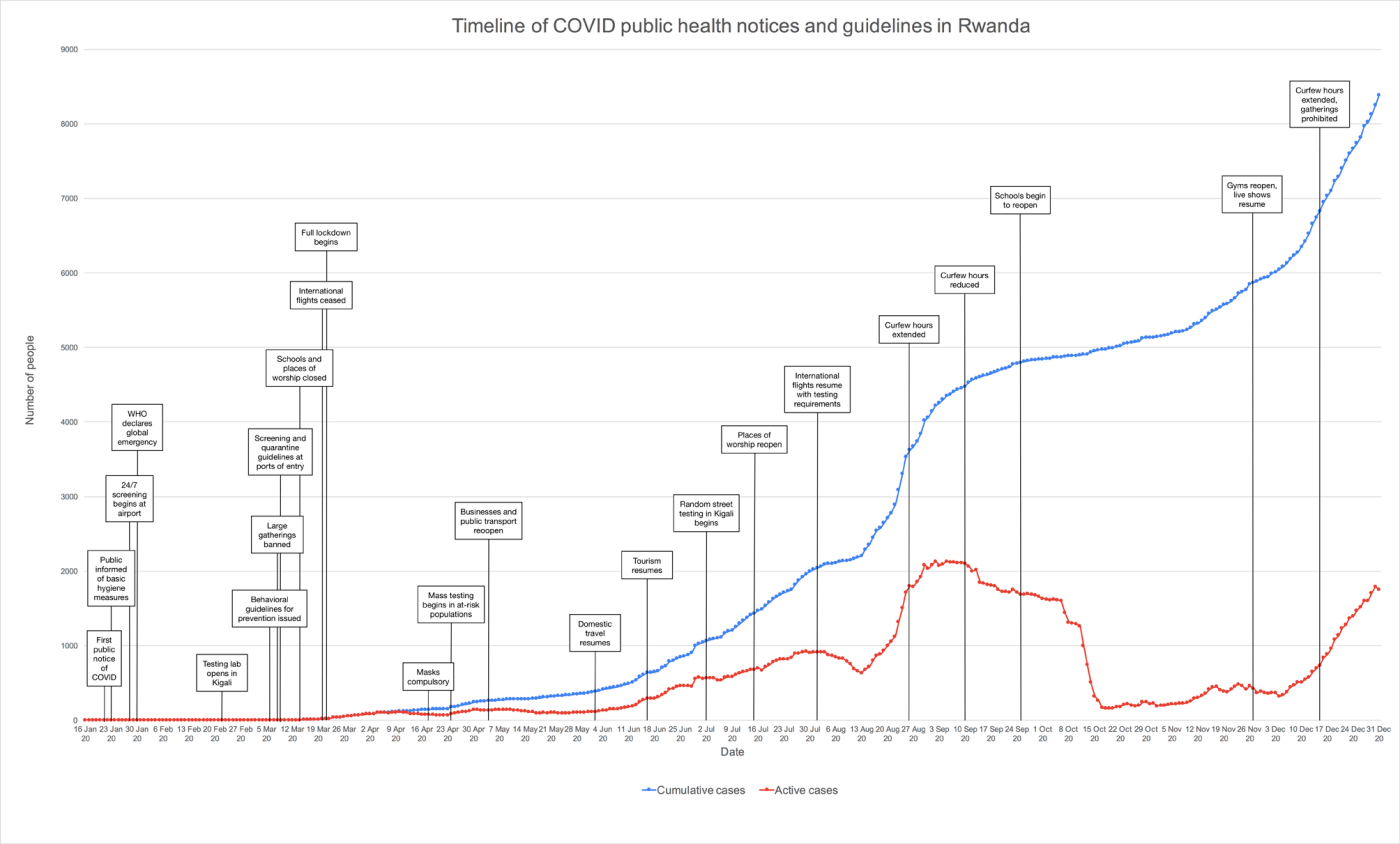
Timeline of official COVID-19 public health notices and guidelines in Rwanda and national case burdens.

Consistent communication and public engagement about COVID-19 has been ongoing since late January (***Figure 3***). As part of the response, the pre-established RBC toll-free community health hotline number was transitioned to a COVID-19 hotline, and a dedicated WhatsApp™ number has been established for reporting suspected cases, which will inform the district’s RRT to investigate [21]. Due to the importance of maintaining public trust and confidence during the pandemic, a Risk Communication and Community Engagement (RCCE) team was created to provide a unified source of COVID-19 related information through governmental websites and social media in order to combat misinformation [12]. Mass media campaigns have been heavily used to increase social awareness of the pandemic, a strategy that Rwanda has also previously employed effectively in its preparation for Ebola virus disease [22]. Social media has played a prominent role, with the MOH, Prime Minister, RBC, and other government offices releasing official information on Twitter™, a medium that is already frequently used for public communication in Rwanda. The daily updates also contain reminders of hygiene and prevention practices and emphasize important new public health guidelines, which are usually first announced by the Prime Minister [23]. Importantly, official Twitter accounts also promptly acknowledge and debunk inaccurate rumors circulating on social media [16].

Local governance and stakeholders have been increasing public awareness for COVID-19 prevention using public and private media, as well as mobile sounds systems via drones equipped with speakers that describe COVID-19 symptoms. Prevention measures, including hand washing and avoidance of handshake greetings, have been promoted through this approach as well. Communication as part of the pandemic management has been strengthened and controlled by the communication cell of the COVID-19 Joint Task Force Coordination responsible for awareness and community engagement, media management, rumors management [12].

The MOH first alerted the public to the presence of COVID-19 in Asia on 21 January [16]. In the following week, the MOH released basic hygiene advice for the public and has since regularly posted updates on the nation’s COVID prevention and response measures [16]. Initial infection control measures were implemented on 28 January, more than a month before Rwanda’s first confirmed case in March. These measures included temperature screening and symptom assessment of travelers at all national points of entry, as well as protocolized screening methods within communities and healthcare facilities [21]. Beginning with the first case on 14 March, daily updates have been released about the number of new cases and their locations (identified in the first month as either a traveler, result of contact tracing, or case of community transmission), recoveries and deaths, currently active cases, and the number of tests conducted [16].

After the first confirmed case, the MOH announced the closure of all schools until September 2020, places of worship, and nightclubs; the postponement of weddings and sporting events; and the use of 114 toll-free number to report suspected COVID symptoms [16]. In the following week, guidelines for controlling the quality and price for masks, sanitizers, and food were released. All arriving and departing commercial flights were ceased for an initial period of 30 days, and mandatory 14-day quarantines were implemented for recent travelers [16]. Due to the continued rise of cases, on 21 March the Office of the Prime Minister issued sweeping infection control measures to promote social distancing through various measures, including restrictions on non-essential travel between cities and public transportation, border closures, and non-essential business closures [23]. These lockdown measures were extended through 3 May, with enforcement provided by police officers regulating circulation within and between districts [23]. Government-coordinated relief efforts began in late March, with food distribution to vulnerable families identified by neighborhood leaders in order to address unemployment and food insecurity as a consequence of lockdown measures [24]. On 18 April, the MOH also announced that masks would be compulsory in public, following changes in mask recommendations from the WHO [22]. Following the discovery that cross-border truck drivers delivering essential goods were contributing to a sharp increase in cases on 24 April, protocols for prevention and testing for cargo transport workers at the borders were developed [23,25]. Regional partnership has been essential to Rwanda’s response; for example, the protocols for truck drivers were developed through negotiations with Tanzania, and continued shared guidelines within the East African Community will help Rwanda maintain its control of the pandemic [25,26].

From May to July, Rwanda gradually eased lockdown restrictions. In May, full lockdown ended and a curfew was instated; businesses and transport within Kigali resumed and hotels and restaurants were required to register all patrons to facilitate contact tracing [23]. In June, travel between provinces resumed and Rwanda’s national parks reopened for tourism [23]. Places of worship began to reopen in July, and multiple random street testing campaigns have been executed in Kigali to verify the prevalence of COVID-19 following the easing of restrictions [23]. International flights resumed on August 1, 2020. Currently, a negative PCR COVID-19 test certificate must be presented for a test taken no more than 120 hours before a traveler’s flight. Moreover, upon arrival, travelers are retested at the airport and taken to a quarantine hotel of their choice until the test results negative within 24 hours [23]. Schools began to reopen in September, followed by gyms and live entertainment in November [23]. Provinces and villages in Kigali in which new clusters of COVID are found are periodically placed back on lockdown, and testing continues in high risk populations and locations [16]. The public health guidelines are reviewed approximately every two weeks, and regulations are lifted or reinstated and curfew is adjusted based on the pandemic’s progress [23].

## Conclusion

Thus far in the pandemic, Rwanda has one of the lowest incidence rates of COVID-19 infection on the African continent. This is a testament to the country’s early planning and aggressive use of innovative strategies. Rwanda formed a six month National COVID-19 Preparedness and Response Plan that detailed protocols for early testing, contact tracing, community health workers outreach, strengthening communication with the public, screening of travelers at ports of entry, and identifying and assessing countries’ hot spots [27]. The use of social media platforms for public communication, drones/robots to disseminate accurate information, and national toll free numbers have been key successful public health responses [9]. Rwanda was able to begin easing lockdown restrictions on May 4, 2020 and has continued to lift or reinstate restrictions based on continuous risk assessment [23]. Although the trajectory of this virus is hard to predict due to its novelty, innovative strategies used in Rwanda may also be beneficial in other settings.
